# Preoperative anxiety and associated factors among adult surgical patients in Debre Markos and Felege Hiwot referral hospitals, Northwest Ethiopia

**DOI:** 10.1186/s12871-018-0619-0

**Published:** 2018-10-30

**Authors:** Henok Mulugeta, Mulatu Ayana, Mezinew Sintayehu, Getenet Dessie, Tesfu Zewdu

**Affiliations:** 1 Department of Nursing, College of Health Science, Debre Markos University, P.O. Box 269, Debre Markos, Ethiopia; 2 Department of Public Health, College of Health Science, Debre Markos University, P.O. Box 269, Debre Markos, Ethiopia; 3 Department of Nursing, School of Health Science, College of Medicine and Health Science, Bahir Dar University, P.O. Box 79, Bahir Dar, Ethiopia; 4 Department of Nursing, College of Health Science, Assosa University, P.O. Box 18, Assosa, Ethiopia

**Keywords:** Anxiety, Major surgery, Preoperative anxiety, Ethiopia

## Abstract

**Background:**

Anxiety during the preoperative period is the most common problem with a number of postoperative complications such as an increase in postoperative pain, delay of healing and prolong the hospital stay. Further, patients with a high level of preoperative anxiety require higher doses of anesthetic agents and recover poorly. Despite its serious health complications, its magnitude and associated factors have been poorly explored in Ethiopia particularly in the selected study areas.

**Objective:**

To assess preoperative anxiety and associated factors among adult surgical patients in Debre Markos and Felege Hiwot Referral Hospitals, Northwest Ethiopia.

**Method:**

An institution based cross-sectional study was conducted on 353 patients scheduled for surgery using a systematic random sampling technique. The data were collected using the state version of the state-trait anxiety inventory scale. All collected data were entered into Epi-Data version 3.1 and analysis was done by using SPSS version 20 software. Binary logistic regression was performed to assess the effect of independent variables on the dependent variable. A *p*-value < 0.05 was considered as statistically significant.

**Results:**

Overall, 61% (95%CI (55.5–65.7)) patients had significant high level of preoperative anxiety. The most common reported factor responsible for preoperative anxiety was fear of complications 187(52.4%). There was a statistically significant high level of pre-operative anxiety among female patients [AOR 2.19 (95%CI (1.29–3.71))] and patients who lack preoperative information [AOR 2.03(95%CI (1.22–3.39))].

**Conclusion:**

The prevalence of preoperative anxiety was high. The level of preoperative anxiety significantly associated with sex, preoperative information provision, and previous surgical experience. Preoperative psychosocial assessment should be incorporated into a routine nursing practice and every patient should be provided with preoperative information before surgery.

## Background

Anxiety is defined as a feeling of unease, worry, fear, tension, and apprehension. It is a response to external or internal stimuli that can have behavioral, emotional, cognitive, and physical symptoms [1]. Perioperative period is one of the worrying events for most surgical patients. It often triggers emotional, cognitive and physiological responses. The goal of perioperative nursing care is to provide better environments and quality of life of a patient before, during and after operation [2].

Preoperative anxiety is a challenging problem in the preoperative care of patients. A common low level of anxiety is an expected reaction to the unpredictable and potentially life-threatening circumstances, especially for a patient’s first surgical experience. However, higher and extended level of preoperative anxiety results in a delay in wound healing as well as requires larger doses of anesthetics and recover poorly. Most patients in the preoperative phase experience anxiety and it is commonly considered as a usual patient response [3–5].

Preoperative anxiety has a number of postoperative complications on the patient, and one of these complications is pain. Pain is the common complaint of post-operative patients which is mostly occur due to preoperative anxiety as a common factor. Pre-operative anxiety has been found to lead to a number of problems such as nausea, vomiting, cardiovascular disturbances such as tachycardia and hypertension, and increased the risk of infection. Studies also showed that a large proportion of surgical patients experience considerable preoperative anxiety and this reported to affect 60–80% of surgical patients [6–8].

The degree to which each patient manifests anxiety depends on many factors such as the patient’s susceptibility to preoperative anxiety, age, gender, past experiences with the surgery, educational status, type and extent of the proposed surgery, current health status, and socioeconomic status. Identifying risk factors helps the nurse to provide psychological support during the preoperative visit so that stress can be reduced. Some group of patients, for example, females, younger patients, patients who have no previous history of surgical operation experience have an increased level of preoperative anxiety [9–12].

A study done in Canada, Saudi Arabia, and Sri Lanka showed that the overall prevalence of preoperative anxiety was 89%, 55%, and 76.7%, respectively [11, 13, 14]. Similarly, a study conducted in Austria reported that the overall preoperative anxiety was 45.3% among admitted surgical patients [15]. Moreover, the result of a study done in a tertiary hospital in Nigeria and a pilot study in Niger showed that 51.0% and 90% of surgical patients had significant preoperative anxiety respectively [16, 17].

In Ethiopia, a limited study has been conducted on preoperative anxiety among surgical patients. In a recent study conducted in South Western Ethiopia, 70.3% of preoperative patients had significant preoperative anxiety [18]. The magnitude of preoperative anxiety among adult surgical patients in the Ethiopian population is not well-known yet. Therefore, the objective of this study was to assess the prevalence of preoperative anxiety and associated factors among adult surgical patients in Debre Markos and Felege Hiwot Referral Hospitals.

## Methods

### Study design and setting

An institution based cross-sectional study design was conducted in Debre Markos and Felege Hiwot referral hospitals from February 01 to April 30, 2017, on 353 patients scheduled for surgery. Debre Markos Referral Hospital is one of the referral hospitals in the Amhara region which is found in Debre Markos town. Debre Markos town, found in the northwest of Addis Ababa, is the capital city of East Gojjam zone, which is found from 265 km from Bahirdar, the capital city of Amhara national Regional state and 300 km from Addis Ababa, the capital city of Ethiopia. Felege-Hiwot referral hospital is found in Bahir Dar. Bahir Dar is found 558 km far away from the capital city of Ethiopia, Addis Ababa.

### Eligibility criteria

Inclusion criteria: All adult elective major surgical Patients age > =18 years.

Exclusion criteria: Patients have known psychiatric illness and those who were on any type of anxiolytic medications. Critically ill patients who were not sufficiently alert to be able to respond to questions.

### Sample size and sampling technique

The sample size was determined by using a single proportion for a finite population with assumptions of 95% confidence level, marginal error (d) of 5% and the prevalence (P) of 70.3% [18]. So, this proportion was used to determine the sample size.

$$ \mathbf{n}=\frac{{\left(\mathbf{Z}\boldsymbol{\upalpha } /\mathbf{2}\right)}^{\mathbf{2}}\mathbf{P}\left(\mathbf{1}\hbox{-} \mathbf{P}\right)}{{\mathbf{D}}^{\mathbf{2}}} $$

**Where:** n = the desired sample size

Za/2 = standard normal score (at 95% confidence level)

**Where: Z = 1.96**

P = prevalence (70.3%)

D = degree of accuracy desired (5%)

$$ {\displaystyle \begin{array}{c}\mathbf{n}=\frac{{\left(\mathbf{1}.\mathbf{96}\right)}^{\mathbf{2}}\left(\mathbf{0.703}\right)\left(\mathbf{1}\hbox{-} \mathbf{0.703}\right)}{{\left(\mathbf{0.05}\right)}^{\mathbf{2}}}\\ {}=\mathbf{321}+\mathbf{Non}\hbox{-} \mathbf{responsive}\kern0.17em \mathbf{rate}\left(\mathbf{1}\mathbf{0}\%\right)\\ {}=\mathbf{353}\;\mathbf{major}\kern0.17em \mathbf{surgical}\kern0.17em \mathbf{patients}\end{array}} $$

Samples were selected from the two referral hospitals (Debre Markos referral hospital and Felege Hiwot referral hospital) in Northwest Ethiopia. Then the total number of adult elective major surgical patient admission from February to April 2016 (last year) was taken to estimate the current admission and the total sample size from each hospital calculated by using a proportion to size allocation formula. Then a systematic random sampling technique was employed to approach the study participants. The sampling fraction (Kth) was calculated by dividing N/n (753/353 = 2). Accordingly, every other patient was selected to participate in the study until the required sample size was achieved.

### Variables

Dependent Variable: Preoperative anxiety (High/low).

Independent Variables: Socio-demographic characteristics, previous surgical experience, previous hospitalization, preoperative information, awareness of the disease and awareness of the type of surgical procedure.

### Operational definitions and definition of terms

**Adult:** a person who is greater than 18 years old.

**Surgery/ Operation:** a procedure that involves cutting a patient’s tissues [18].

**Anxiety:** is a vague feeling of dread or apprehension [1].

**Level of anxiety:** - expressed by a score of S- STAI as high-level anxiety and low-level anxiety [16].

**High-level anxiety:** - Patient who score S- STAI > 44 [16].

**Low-level anxiety:** - Patient who score S- STAI ≤44 [16].

**Major surgery:** any surgical operation that is performed in the major operating room using spinal or general anesthesia. Example: Cholecystectomy, hemorrhoidectomy, hernia repair, thyroidectomy, orthopedic surgeries, appendectomy, prostatectomy, colon surgeries.

### Data collection and instruments

The data were collected using validated and standardized preoperative anxiety measuring tool that is State Version (Y-1) of State-Trait Anxiety Inventory Scale (S-STAI), which is adapted from other studies [16, 18], with some modification to increase the comparability of the finding. The questions and statements were grouped and arranged according to the particular objectives that can address based on experts’ comments. The STAI Form Y is the absolute instrument for measuring preoperative anxiety in adults. The questionnaire was prepared in English and it was translated into the local language, Amharic and back to English for consistency.

State-Trait Anxiety Inventory Scale (S-STAI) is a self-report measure that has two subscales. First, the State Anxiety Scale (S-Anxiety) evaluates the current state of anxiety, asking how respondents feel “right now,” using items that measure subjective feelings of apprehension, tension, nervousness, worry, and activation/ arousal of the autonomic nervous system. The Trait Anxiety Scale (T-Anxiety) evaluates relatively stable aspects of “anxiety proneness,” including general states of calmness, confidence, and security. Reliability and validity of the STAI are well reported (Cronbach’s alpha = 0.86) and measurement of state anxiety is recommended in the perioperative period.

The STAI has 40 items, 20 items allocated to each of the S-Anxiety and T-Anxiety subscales. Responses for the S-Anxiety scale assess the intensity of current feelings “at this moment”: 1) not at all, 2) somewhat, 3) moderately so, and 4) very much so. Responses for the T-Anxiety scale assess the frequency of feelings “in general”: 1) almost never, 2) sometimes, 3) often, and 4) almost always. In the state portion of STAI (Y-1), ten statements express anxiety (item number 3, 4, 6, 7, 9, 12, 13, 14, 17 and 18) while the remaining 10 statements (item number 1, 2, 5, 8, 10, 11, 15, 16, 19 and 20) represent the relaxed and pleasant state of patient [19].

A rating of 4 indicates the presence of high level of anxiety for ten S-Anxiety items and a high rating indicates the absence of anxiety for the remaining ten S-Anxiety items. The scoring weights for the anxiety-absent items are reversed. The scores of STAI range from a minimum of 20 to a maximum score of 80. The score of more than 44 on STAI was taken as significant anxiety and patient was categorized as high anxiety (STAI score > 44) while low anxiety (STAI score ≤ 44). The selection of this value was based on previously published researches [16, 18–22].

The data were collected through interview using structured questionnaire by trained four BSc nurses and the data collection took only 20 min to complete for one patient. Two Supervisors were closely supervised the process of data collection.

### Data quality control

To assure the quality of data training about the questionnaire was given to data collectors for one day prior to data collection, a pretest was done on 10% of the sampled population. Collected data were checked for its completeness and clarity on daily basis and corrections were made accordingly. Follow up and supervision was also conducted by supervisors during data collection.

### Data processing and analysis

The collected data were entered into Epi-Data version 3.1 and exported to SPSS version 20 for data cleaning and analysis. Descriptive analysis was performed to describe the number and percentage of socio-demographic characteristics of the sample and other variables. The binary logistic regression model was fitted to estimate the effect size of independent variables on the dependent variable. Odds ratio with its 95% confidence interval was used to estimate the strength of association. First, a bivariate analysis was computed to test the association between each independent variable with the dependent variable. In bivariable analysis, all independent variables with a *p*-value of less than 0.2 were included for further analysis in the multivariable regression model. Then to control for possible confounders multivariable backward logistic regression was performed to explore the risk of “high anxiety” associated with the patient characteristics. A *p*-value of 0.05 or below was considered to declare statistically significant association or effect. The results were presented in text, tables, and graphs based on the types of data.

## Results

### Socio- demographic characteristics

A total of 353 elective major surgical patients participated in the study with a response rate of 100%. One hundred Eighty-two (51.6%) of the participants were males and the majority (61.2%) belong to the age of 18–39 years with a median age of 40 (IQR = 28.5–50) years. The result of this study showed that 135(64.6%), 73(20.7%), 24(6.8%), 19(5.4%), and 9(5%) were married, single, widowed, divorced, and separated respectively. The ethnic and religious composition of the patients showed that322 (91.2%) were Amhara and 307 (87%) were Orthodox Christians. Among all respondents, 188(53.3%) did not attend formal education. The occupational status of the participants showed that 124(35.1%) were farmers, 49(13.9%) were merchants, 49(13.9%) were housewives, 65(18.4%) were private workers, 35(9.9%) were a governmental employee and 31(8.8%) were students. Out of the total respondents, 208(59%) came from rural areas**.** Socio-demographic characteristics summarized in Table 1.

**Table 1:** Socio demographic characteristics of surgical patients in the referral hospitals

Variables	Response	Frequency (n)	Percentage (%)
Age (in Years)	18–39	168	47.6
	40–49	88	24.9
	50–59	44	12.5
	> 60	53	15
Sex	Male	182	51.6
	Female	171	48.4
Educational status	Not attended formal education	188	53.3
	Primary education (1–8)	74	21
	Secondary education (9–12)	49	13.9
	College and above	42	11.9
Religion	Orthodox	307	87
	Muslim	26	7.4
	Protestant	17	4.8
	Catholic	3	0.8
Ethnicity	Amhara	322	91.2
	Oromo	18	5.1
	Tigre	13	3.7
Residence	Urban	208	59
	Rural	145	41
Income (in ETB)	< 1000	121	34.3
	1001–2500	96	27.2
	2501–3999	56	15.9
	> 4000	80	22.7
Family size	< 3	91	25.8
	4–6	184	52.1
	> 6	78	22.1

### Previous health status history of the patient

Among all respondents, 144(40.8%) had a history of previous hospitalization and 61(17.3%) had previous surgical operation experience. Past health status condition of surgical patients was presented in Table 2.

**Table 2:** Past health status condition of surgical patients the referral hospitals

Variables	Responses	Frequency	Percentage
Previous hospitalization	Yes	144	40.8
	No	209	59.2
Previous surgical operation	Yes	61	17.3
	No	292	82.7
Time of the previous operation (in years)	< 1 year	10	2.8
	2–5 years	30	8.5
	> 6 years	7	2
Time of the previous operation (in months)	< 5	9	2.5
	> 5	5	1.4
Frequency of the previous operation performed	Once	50	14.2
	Twice	10	2.8
	Thrice or more	1	0.3
Any previous surgery complications	Yes	9	2.5
	No	52	14.7
Preoperative information during the previous surgery	Yes	22	6.2
	No	24	6.8
	I don’t remember	15	4.2

### Current health status history of the study participants

Majority of the total study participants 229(64.9%) know their diagnosis and 191(54.1%) had knowledge of the type of surgery to be performed. Respondents were asked whether they had got preoperative information or not. Among those, 179(51%) of them were not provided with appropriate preoperative information about the surgical operation and preoperative anxiety. Current health status history of the study participants was summarized in Table 3.

**Table 3:** Awareness of study participants on their current health status and Preoperative information provision

Variables	Responses	Frequency	Percentage
Know the disease (diagnosis)	Yes	229	64.9
	No	124	35.1
Know the type of surgery to be performed	Yes	191	54.1
	No	162	45.9
Presence of pain on admission	Yes	122	34.6
	No	231	65.4
Preoperative information provision	Yes	174	49
	No	179	51
Satisfaction with information provided	Yes	129	36.5
	No	45	12.7

**Fig. 1:**
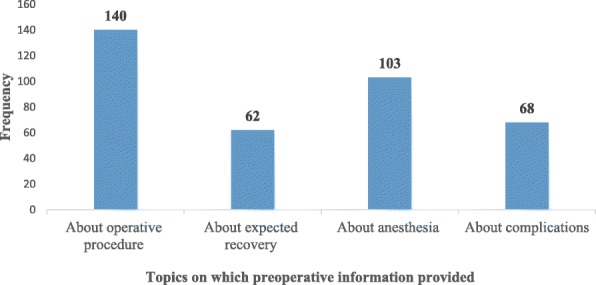
Preoperative information topics for surgical patients in the referral hospitals

Among those who had got preoperative information, 140(39.7%) respondents got information only about operative procedures, while 17 respondents got information about operative procedures expected recovery, anesthesia and surgery complications (Fig. 1).

### Prevalence of preoperative anxiety

The prevalence of preoperative anxiety (state anxiety) was assessed using the state version of the State-Trait Anxiety Inventory scale (STAI). Overall 61% (95%CI (55.5–65.7)) of surgical patients in this study had significant high level of preoperative anxiety (having S-STAI scores above 44). The median score for state-anxiety (S-STAI) was 49 with IQR of 38.5–56.5. Concerning the state anxiety items, 141(39.9%) of patients scored moderately calm with a median score of 2 with IQR of 2–3. STAI state (Y-1) anxiety score of surgical patients was presented in Table 4.

**Table 4:** STAI state (Y-1) anxiety score of surgical patients in referral hospitals

S. No	Variables	Not at all N (%)	Somewhat N (%)	Moderately N (%)	Very much N (%)
1	I feel calm^a^	36 (10.2)	155 (43.9)	141 (39.9)	21 (5.9)
2	I feel secure^a^	45 (12.7)	150 (42.5)	131 (37.1)	27 (7.6)
3	I feel tense	113 (32)	150 (42.5)	67 (19)	23 (6.5)
4	I am strained	47 (13.3)	189 (53.5)	93 (26.3)	24 (6.8)
5	I feel at ease^a^	70 (19.8)	165 (46.7)	103 (29.2)	15 (4.2)
6	I feel upset	178 (50.4)	95 (26.9)	67 (19)	13 (3.7)
7	I am presently worrying…	86 (24.4)	168 (47.6)	84 (23.8)	15 (4.2)
8	I feel satisfied^a^	57 (16.1)	187 (53)	93 (26.3)	16 (4.5)
9	I feel frightened	75 (21.2)	185 (52.4)	75 (21.2)	18 (5.1)
10	I feel comfortable^a^	74 (21)	169 (47.9)	105 (29.7)	5 (1.4)
11	I feel self-confident^a^	22 (6.2)	138 (39.1)	137 (38.8)	56 (15.9)
12	I feel nervous	122 (34.6)	152 (43.1)	64 (18.1)	15 (4.2)
13	I feel jittery	122 (34.6)	172 (48.7)	48 (13.6)	11 (3.1)
14	I feel indecisive	146 (41.4)	157 (44.5)	44 (12.5)	6 (1.7)
15	I am relaxed^a^	110 (31.2)	143 (40.5)	93 (26.3)	7 (2)
16	I feel content^a^	113 (32)	151 (42.8)	74 (21)	15 (4.2)
17	I am worried	44 (12.5)	201 (56.9)	73 (20.7)	35 (9.9)
18	I feel confused	138 (39.1)	137 (38.8)	56 (15.9)	22 (6.2)
19	I feel steady^a^	83 (23.5)	140 (39.7)	114 (32.3)	16 (4.5)
20	I feel pleasant^a^	104 (29.5)	153 (43.3)	75 (21.1)	21 (5.9)

Note: ^a^ Positive questions which are reverse coded

### Reported factors responsible for preoperative anxiety among surgical patients

There are various responsible factors for preoperative anxiety in patients undergoing a surgical operation. In this study, among the factors responsible for preoperative anxiety, the most common reported factors responsible for preoperative anxiety were fear of complications 187(52.4%), concern about family 178(50.4%), fear of postoperative pain 177(50.1%) and fear of death 170(48.2%). Reported factors responsible for preoperative anxiety among surgical patients were presented in Table 5.

**Table 5:** Factors responsible for preoperative anxiety among surgical patients in the referral hospitals

Variables	Responses		State anxiety score Median (IQR)
	Frequency (N)	Percent (%)	
Fear of complications	185	52.4	50 (39–58)
Concern about family	178	50.4	49 (37–56)
Postoperative pain	177	50.1	49 (40–56)
Fear of death	170	48.2	51 (41–59)
Change of environment	160	45.3	57 (41–61)
Results of operation	150	42.5	49 (39–56)
Harm from doctor/nurse mistake	107	30.3	53 (46–59)
Fear of unknown	104	29.5	50 (40–59)
Fear of physical disability	96	27.2	53 (47–61)
Waiting for operation	65	18.4	48 (39–54)
Financial loss	62	17.6	53 (49–61)
Nil per mouth	60	17	50 (39–60)
Awareness during surgery	60	17	52 (39–60)

Note: Proportion (%) cannot be 100% because it is based on multiple response questions

### Statistical analysis of factors associated with preoperative anxiety

In bivariable analysis, the factors with a *p*-value of less than 0.20 were sex, age, marital status, educational level, occupation, residence, family size, preoperative information, and previous surgical experience. However, those variables such as awareness of the diagnosis, awareness of the type of surgery to be performed and previous hospitalization did not show any significant association up to *P*-value of 0.2 so that subsequently were not included in the final model.

Multivariable backward logistic regression analysis showed that a model with the five independent variables: sex, educational level, preoperative information provision, and previous surgical experience, family size was significantly associated with preoperative anxiety. The odds of having preoperative anxiety was 2.19 (95%CI (1.29–3.71)) times higher among females as compared to males (Table 6).

**Table 6:** Bivariable and Multivariable logistic regression analysis of factors associated with preoperative anxiety

VARIABLES	Level of anxiety		COR (95%CI)	AOR (95%CI)
	High (%)	Low (%)		
Sex				
Male	83 (38.8)	99 (71.2)	1	1
Female	131 (61.2)	40 (28.8)	3.91 (2.47–6.18)	2.19 (1.29–3.71)
Educational level				
Not attended formal Edu.	126 (58.9)	62 (44.6)	8.64 (3.77–19.77)	7.06 (2.88–17.29)
Primary education (1–8)	54 (25.2)	20 (14.4)	11.48 (4.55–28.95)	7.07 (2.63–19.02)
Secondary education (9–12)	26 (12.1)	23 (16.5)	4.80 (1.85–12.46)	5.04 (1.81–13.99)
College and above	8 (3.7)	34 (24.5)	1	1
Preoperative information				
Yes	87 (40.7)	87 (62.6)	1	1
No	127 (59.3)	52 (37.4)	2.44 (1.58–3.79)	2.03 (1.22–3.39)
Previous surgical experience				
Yes	19 (8.9)	42 (30.2)	1	1
No	195 (91.1)	97 (69.9)	4.44 (2.45–8.05)	3.05 (1.57–5.95)
Family size				
< =3	66 (30.8)	25 (18)	2.15 (1.13–4.08)	3.21 (1.50–6.90)
4–6	105 (49.1)	79 (56.8)	1.08 (0.64–1.84)	1.23 (0.67–2.27)
> 6	43 (20.1)	35 (25.2)	1	1

## Discussion

The overall prevalence of preoperative anxiety in this study was 61% (95%CI (55.5–65.7)) as suggested by STAI score of more than 44 which showed that most of the patients awaiting elective surgery experienced a high level of preoperative anxiety. This result was similar to the previous study conducted among Pakistan surgical patients using a similar tool in which the overall prevalence of preoperative anxiety was 62%(STAI score of more than 44) [22]. In parallel, similar results were seen from another study conducted in Indian surgical patients using a different tool, where the overall prevalence of preoperative anxiety was 58.9% [23].

The result of this study found to be higher than another study conducted in Austria, Saudi Arabia and Nigeria where the overall prevalence of preoperative anxiety among admitted surgical patients was 45.3% and 55% and 51% respectively [14–16]. This might be due to the fact that most of the study participants in this study were poor and lower-class patients with poor educational status so that they had no access to information regarding anesthesia and the surgical procedure they were about to undergo.

The prevalence of preoperative anxiety in this study is lower than other similar studies conducted in Canada, Seri Lanka and Niger where the overall prevalence was 89%, 76.7%, 90% respectively [11, 13, 17]. The possible reason for the lower proportion of anxiety in this study might be due to strong family and social support implemented in our society. In addition, the difference could be due to methodological issues and measurement tool used to quantify the level of preoperative anxiety. Furthermore, the prevalence of anxiety in this study is lower than other similar study conducted among surgical patients in Jimma University Specialized Teaching Hospital, South Western Ethiopia [18]. This might be due to a difference in socio-demographic characteristics of the study participants.

There are various factors responsible for preoperative anxiety in patients undergoing a surgical operation. In this study, the most common responsible factor for preoperative anxiety was fear of complications. This was in line with a research done in Nigeria [16]. Concern about family and fear of postoperative pain in this study were the second and third most common responsible factors respectively. This was inconsistent with another study where concern about family and fear of postoperative pain were ranked the first and the second common responsible factors for preoperative anxiety respectively [24]. A study done in check republic demonstrated that their patient’s most common cause of preoperative anxiety was fear of postoperative pain while in this study it was ranked third [25]. Similarly, fear of death in this study was the fourth top responsible factor, but it was the most common cause of anxiety in another study [18].

The socio-demographic characteristics that were significantly associated with preoperative anxiety were sex and educational level. The findings of this study showed that female patients had a statistically significant higher level of preoperative anxiety than males. These associations have also been demonstrated by previous similar studies [13, 17, 23–27]. The difference could be because women are sensitive to fearful events and differences in hormone fluctuations. In addition, females more easily express their anxiety than men, and separation from the family affects women more. However, one study showed no association between sex and preoperative anxiety [28].

In this study history of previous surgical experience was a significant factor for preoperative anxiety. Patients with a history of previous surgical experience were less anxious than patients coming for surgery for the first time. This was in line with other similar studies [13, 25]. This could be because of less fear of surgery or less misunderstandings about anesthesia and surgery. Contrary to this, a number of studies showed that a history of previous surgical experience and level of preoperative anxiety were not significantly associated [18, 22–24].

Another finding of this study was the level of education were significantly associated with the level of anxiety. In this study, the level of anxiety decreases with increasing level of education. This was consistent with another similar study [29]. This could be because increase level of education helps patients in preparing and reducing anxiety preoperatively. In addition, a larger proportion of anxious patients with lower education level may be because of their poor awareness related to anesthesia and surgery. Contrary to this, the results of other similar studies revealed that the level of preoperative anxiety appeared to increase with increasing level of education [18, 22, 27].

The findings of this study showed that patients who had information regarding the surgical procedure and anesthesia had a lower state-anxiety score. A marked decrease was observed in the state anxiety score of patients who had preoperative information. This was in line with many other studies [7, 18, 28, 30].

## Limitation of the study

Although this has provided valuable information regarding the prevalence of preoperative anxiety and associated factors among surgical patients, there were some limitations that could be addressed in future research. First, this study did not measure patients’ anxiety level before admission. Second, Comparison of the level of anxiety preoperatively and postoperatively among respondents was not done. Lastly, the Pediatric group was excluded.

## Conclusion

In this study, the prevalence of preoperative anxiety was high. The level of preoperative anxiety significantly associated with sex, educational level, preoperative information provision, and previous surgical experience. In addition, Fear of complications, concern about family and fear of postoperative pain were the most common factors responsible for preoperative anxiety. Preoperative psychosocial assessment should be incorporated into a routine preoperative nursing practice and adequate, and appropriate preoperative information should be provided for surgical patients before surgery.

## Abbreviations

AOR: Adjusted Odds Ratio
APAIS: Amsterdam Preoperative Anxiety and Information Scale
CI: Confidence Interval
COR: Crude Odds Ratio
HADS: Hospital Anxiety and Depression Scale
IQR: Interquartile Range
JUSH: Jimma University Specialized Hospital
SPSS: Statistical Package for Social Sciences
STAI: State-Trait Anxiety Inventory
VAS: Visual Analog Scale
